# Left atrial appendage sizing for percutaneous closure in virtual reality—a feasibility study

**DOI:** 10.3389/fcvm.2023.1188571

**Published:** 2023-09-01

**Authors:** Houtan Heidari, Dominika Kanschik, Ralf Erkens, Oliver Maier, Georg Wolff, Raphael Romano Bruno, Nikos Werner, Sebastian Daniel Reinartz, Gerald Antoch, Malte Kelm, Tobias Zeus, Christian Jung, Shazia Afzal

**Affiliations:** ^1^ Division of Cardiology, Pulmonology and Vascular Medicine, Medical Faculty, Heinrich Heine University, Düsseldorf, Germany; ^2^ Heartcenter Trier, Krankenhaus der Barmherzigen Brüder, Trier, Germany; ^3^ Institute of Diagnostic and Interventional Radiology, Heinrich Heine University of Düsseldorf, Düsseldorf, Germany; ^4^ Department of Diagnostic and Interventional Radiology, Medical Faculty, University Clinic Duesseldorf, Heinrich-Heine University Duesseldorf, Düsseldorf, Germany; ^5^ CARID (Cardiovascular Research Institute Düsseldorf), Heinrich Heine University, Düsseldorf, Germany

**Keywords:** left atrial appendage closure, virtual reality, cardiac computed tomography, device sizing, imaging

## Abstract

**Background and aims:**

The complex and highly variable three-dimensional anatomy of the left atrial appendage (LAA) makes planning and device sizing for interventional occlusion procedures (LAAC) challenging. Several imaging modalities [e.g. echocardiography, multi-slice computed tomography (MSCT)] are used for this purpose. Virtual reality (VR) is an emerging imaging technique to immerse into a three-dimensional left atrium and appendage, offering unprecedented options of visualization and measurement. This study aimed to investigate the feasibility, accuracy and reproducibility of visualizing the LAA in VR for preprocedural planning of LAAC.

**Methods and results:**

Twenty-one patients (79 ± 7 years, 62% male) who underwent LAAC at University Hospital Düsseldorf were included in our study. A dedicated software generated three-dimensional VR models from preprocedural MSCT imaging data. Conventional measurements of LAA dimensions (ostium, landing zone and depth) using a commercially available software were compared to measurements in VR: MSCT and VR ostium min. (*r* = 0.93), max. (*r *= 0.80) and mean (*r *= 0.88, all *p* < 0.001) diameters as well as landing zone (LZ) min. (*r *= 0.84), max. (*r *= 0.86) and mean diameters (*r *= 0.90, all *p* < 0.001) showed strong correlations. Three-dimensional orientation was judged superior by physicians in VR compared to MSCT (*p* < 0.05).

**Conclusion:**

Virtual reality visualization of the left atrium and appendage based on MSCT data is feasible and allows precise and reproducible measurements in planning of LAA occlusion procedures with enhanced 3D orientation. Further studies need to explore additional benefits of three-dimensional visualization for operators in preprocedural planning.

## Graphical Abstract

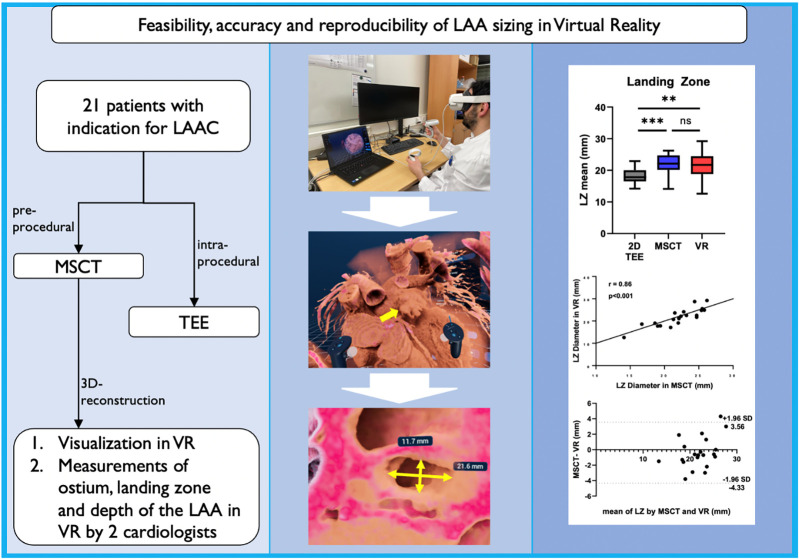

Application of VR for LAA sizing: 21 patients with preprocedural MSCT and intraprocedural TEE were included. LAA measurements performed in the virtual environment showed strong correlation with current gold standard MSCT and were highly reproducible. In addition, VR offered a superior spatial orientation and depth perception. LAA, left atrial appendage; LAAC, left atrial appendage closure; MSCT, multi-slice computed tomography; TEE, transesophageal echocardiography; VR, virtual reality; LZ, landing zone.

## Introduction

Advances in multimodal imaging have been a cornerstone for the evolution of minimally invasive interventional procedures (1). Virtual reality (VR) visualization of the heart is a novel technique in planning of interventional procedures such as left atrial appendage closure (LAAC), promising a better understanding of patient-specific complex anatomy and an increase in precision of depth and scaling (2–4).

LAAC has emerged as a safe and efficient treatment option for stroke prevention in multimorbid patients (5, 6). The LAA is a highly complex and variable anatomical structure, which poses challenges to preprocedural planning and device selection (7). Different imaging modalities such as 3D transesophageal echocardiography and multi-slice computed tomography (MSCT) are used to evaluate LAA morphology and obtain dimensions of ostium and landing zone for optimal device selection, device position and final sealing of LAA. Despite of the fact that MSCT provides better three dimensional understanding precise LAA landing zone prediction remains crucial for procedural success. Subsequent undersizing of occluder devices may result in peri-device leakage or device embolization or occurrence of device-related thrombus, whereas oversizing may pose risk for perforation with consecutive pericardial effusion (8, 9). Minimal and maximal diameters from 3D reconstruction of transesophageal echocardiography (TEE) and/or MSCT are used for preprocedural planning (10), however, these 3D imaging datasets are displayed on regular 2D screens, which limits the diagnostic capabilities of the volumetric datasets. Thus, experience of the interventionalist is required to mentally reconstruct a 3D model in order to accurately plan the procedure and predict device interaction with adjacent structures. Studies using 3D printing for planning of LAAC have shown more accurate device sizing, reduced number of devices used per procedure and a reduced fluoroscopy time (11). VR applications offer the same advantages of a true 3D visualization, however at considerably lower cost.

The aim of our study was to evaluate the feasibility and accuracy of LAA measurements in VR for device sizing in patients undergoing LAAC.

## Methods

### Patient population

From June 2019 to October 2021, all patients with non-valvular atrial fibrillation who underwent LAAC and preprocedural MSCT at University Hospital Düsseldorf were included. All patients received an Amulet Amplatzer^™^ occluder device under conscious sedation and with intraprocedural guidance using TEE and fluoroscopy. All imaging data were retrospectively analyzed. The study was approved by the local ethics committee (no. 5272R) and complies with the declaration of Helsinki.

### Multi-slice computed tomography image acquisition and analysis

All patients underwent pre-procedural high-resolution, contrast-enhanced, electrocardiogram-gated spiral acquisition mode CT (150 ms, 128 × 0.6 mm, Siemens Healthineers, Erlangen, Germany, “SOMATOM Definition Edge”). Images were obtained at 30%–60% of the R-R interval. A delayed scan after contrast injection was used to allow optimal contrast distribution according to established protocols. Images were taken in accordance with LAA-specific expert recommendations on CT acquisition (12, 13). All datasets were saved as Digital Imaging and Communications in Medicine (DICOM) files and processed with dedicated software (3mensio Structural Heart^™^, Pie Medical Imaging BV, Maastricht, The Netherlands). All datasets were evaluated, and measurements performed by an independent physician highly experienced in this modality. Image quality was defined as insufficient in case of inadequate delineation of the endocardial border due to incomplete contrast opacification of the LAA. Furthermore we assessed data set regarding slow flow, sludge or LAA thrombus. In detail, the LAA was analyzed by semi-automated 3mensio LAA workflow. The left circumflex coronary artery and the coumadin ridge were marked and defined the ostium. The landing zone (LZ) was defined at a location 10–12 mm distal from the ostium after adjusting the angle. The depth was measured as a perpendicular line from the ostium to the LAA roof.

### Transesophageal echocardiography (TEE)

Intraprocedural TEE was performed for guidance of the procedure. All TEE studies were performed under conscious sedation by an advanced imaging cardiologist using an EPIQ 7 ultrasound machine and an X8-2T TEE probe (Philips Medical Systems, Amsterdam, Netherlands), as described previously (14). The LAA was visualized in 0°, 45°, 90° and 135° from a midesophageal approach (10). The ostium was defined as a line between tip of the coumadine ridge and the circumflex artery. The LZ was defined as the line 10 mm distal from the ostium perpendicular to the long axis of the LAA. The depth was defined as a straight line from the midpoint of the ostium plane to the LAA roof. Device sizing was based on LZ maximum diameter according to manufacturer's instructions.

### Virtual reality—software and hardware

The Meta Quest 2 (Meta, Irvine, California, USA) VR headset was employed for visualization in VR with Single Fast-Switch LCD display and a resolution of 1,832 × 1,920 pixels per eye with a refresh rate of 72 Hz. Visualization in VR was done using dedicated software (VMersive, Warsaw, Poland) based on MSCT imaging data (DICOM datasets). The software allowed anatomical visualization, including free navigation (6 degrees of freedom, 3 rotational, 3 translational movements) through the virtual heart. Steering the 3D model was enabled with two handheld controllers allowing to zoom, grab, slice, cut out and rotate the model. Distance measurements were directly performed in the VR environment in the same cardiac cycle as measurements in MSCT.

### Workflow and protocol of VR measurements

**Figure 1:**
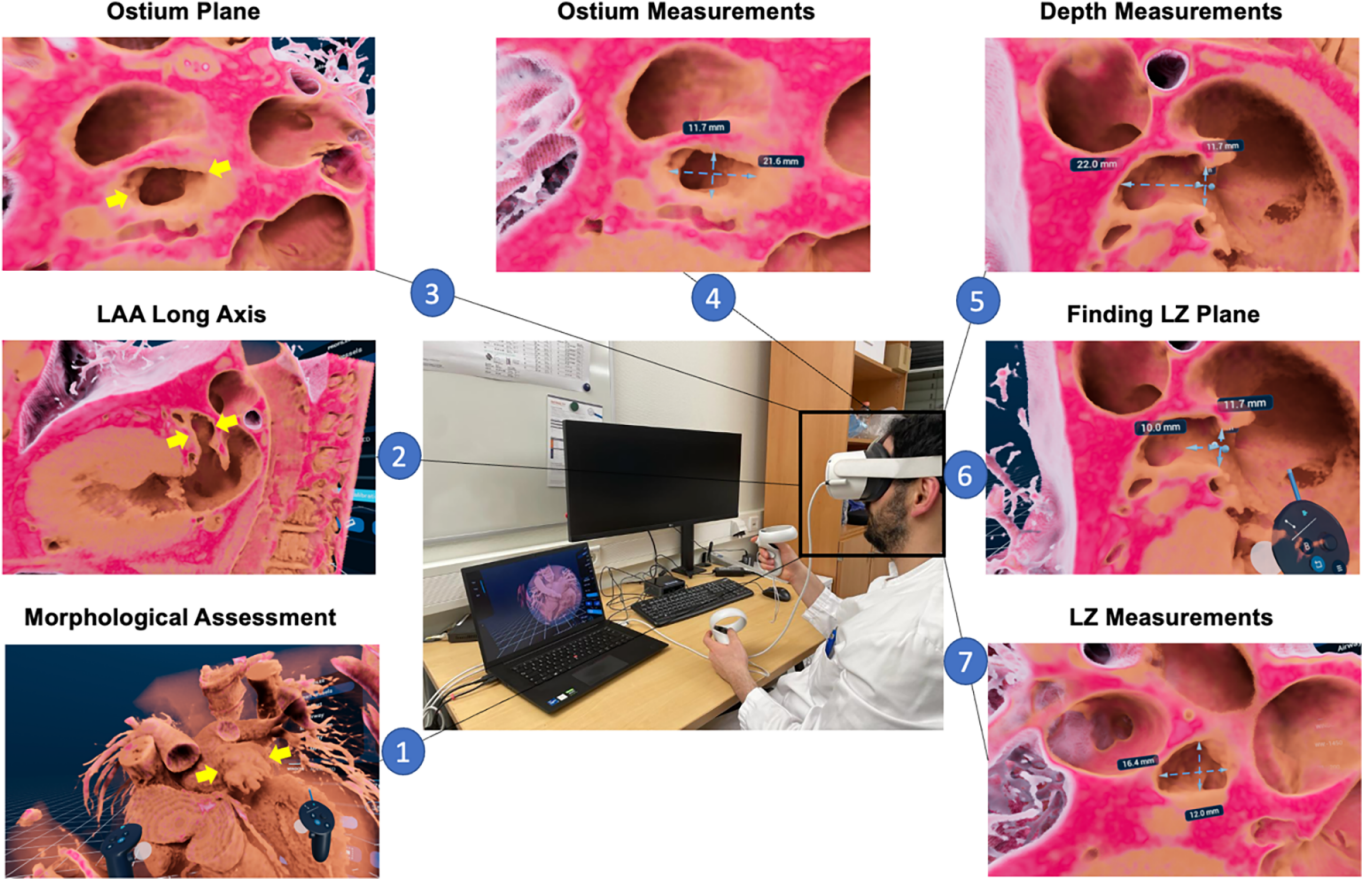
LAA sizing protocol in VR. First, an overview of 3D rendered patent-specific MSCT dataset is displayed in VR environment and the LAA morphology (yellow arrows) can be assessed (1). Next, by cutting through the LAA and the long axis of the left ventricle, the long axis of the LAA is displayed and can visually be assessed (2); subsequently, by cutting through the left atrium perpendicular to the ostium, an en-face view of the LAA orifice is achieved (3), and minimum and maximum diameters can be measured (4); then, the depth from the ostium to LAA roof is measured (5) and at a distance of 10-12 mm distal from the ostium (6) the landing zone minimum and maximum diameters are measured (7); MSCT, multi-slice computed tomography; VR, virtual reality; LAA, left atrial appendage; LZ, landing zone.

The protocol for measurements in VR is displayed in Figure 1: First, high-quality MSCT DICOM datasets were loaded into the VMersive software, which automatically performed 3D reconstruction. Images were visualized in VR and allowed direct and intuitive interaction in the virtual space. In VR, an adequate window setting in the same phase as MSCT measurements with good delineation of the LAA and surrounding structures was selected, which led to an excellent visual display of LAA and its surrounding structures in 3D in all cases. Then, all distantly surrounding structures around the LAA were cut. Here, we specifically appreciated the morphology of the LAA, the direction of the long axis, the number of lobes and the directly surrounding structures to get a comprehensive understanding of the patient-specific anatomy. Next, a specific virtual plane tool was placed to dive through the extracardiac structures to visualize the heart. By placing a plane through the left ventricle and left atrium the LAA was shown anterolateral in virtual space. By diving through the LAA and the long axis of the left ventricle, the long axis of the LAA was achieved and neck and body of LAA was evaluated. Next, by moving through the left atrium perpendicular to the ostium plane, an en-face view of the LAA ostium was achieved and minimum and maximum diameters were measured by a virtual caliper. From the midpoint of the ostium plane, the LAA depth was measured as a straight line to the roof of the LAA. At 10–12 mm, distal from the ostium, the LZ dimensions were measured perpendicular to the long-axis of the LAA (see Figure 1). In addition, nine independent physicians evaluated the workflow and compared three-dimensional orientation and depth perception in LAA visualization comparing MSCT and VR on a five-point Likert scale (with 1 = strongly agree, 2 = agree, 3 = neither disagree, nor agree, 4 = disagree and 5 = strongly disagree).

### Statistics

Continuous variables are displayed as counts and percentages and mean ± standard deviation. The dataset was tested for normal distribution using the D’Agostino-Pearson normality test. Unpaired *t*-test was applied to compare continuous variables. The agreement of MSCT and VR measurements were displayed with Bland-Altman plots. The correlation of MSCT and VR measurements were calculated using Pearson's correlation coefficient. *P*-values < 0.05 were considered statistically significant. All measurements were performed using GraphPad Prism version 9 (GraphPad Software, San Diego, USA).

### Intra- and interobserver variability

All measurements were conducted independently by two experienced imaging specialists to analyse interobserver variability. Inter- and intraobserver variability for VR measurements were calculated using the ICC. One of the investigators repeated the measurements of 10 Patients 3 months after the initial measurement in VR to analyse intraobserver variability. Inter- and intraobserver agreement was considered excellent, good, moderate and poor for ICC >0.90, 0.75–0.90, 0.50–0.75 and <0.50 respectively. The calculation of the intraclass correlation coefficient (ICC) was performed with SPSS (version 28, Chicago, Illinois, USA).

## Results

### Patient population

A total of 21 patients were included in our study. The mean age was 78.5 years (IQR: 74.5–82.3 years). Thirteen patients (62%) were male. Four patients were excluded due to insufficient MSCT image quality. Image quality was defined as insufficient in case of inadequate delineation of the endocardial border due to incomplete contrast opacification of the LAA. This was evaluated by two independent cardiologists. None of the patients included showed slow flow, sludge or LAA thrombus in TEE or MSCT. All patients underwent LAAC with the Amplatzer Amule™ Occluder, 9.5% of patients received an 18 mm occluder, 4.8% a 20 mm occluder, 23.8% a 22 mm occluder, 42.9% a 25 mm and 19% a 28 mm occluder. Overall, few complications occurred in our study cohort. One patient developed arrhythmia, two patients had major and three patients suffered from minor bleedings. No device related complications such as thrombus, device dislocation or pericardial effusion were observed. Further detailed baseline characteristics are displayed in Table 1 and in the Supplementary Material Table S1.

**Table 1:** Patient clinical characteristics.

Baseline characteristics (*n* = 21)	
Mean age (years)	78.5 (74.5–82.3)
Male gender	13 (62%)
Height (in meter)	1.72 (±0.1)
Weight (in kg)	75 (±13.2)
Mean BMI	25.2 (±4.4)
Heart failure	66.7%
CKD	33.3%
Atrial fibrillation	100%
CCS	42.9%
Hypertension	66.7%
Diabetes mellitus	42.9%
Previous PCI	38.1%
Dyslipidemia	47.6%
COPD	0%
NYHA (mean)	1.3 (±1.1)
CHA2DS2-VASc score (mean)	4.7 (±1.8)
Indication for LAAC	
Contraindication for OAC	57.1%
Gastrointestinal bleeding	14.3%
Other bleeding	19%
Labile INR	9.5%
Implanted device size	
18 mm	2 (9.5%)
20 mm	1 (4.8%)
22 mm	5 (23.8%)
25 mm	9 (42.9%)
28 mm	4 (19%)

Values are presented as mean ± SD or expressed in *n* (%).

Overview of clinical patient characteristics, indication and device size for LAAC BMI, body mass index; CKD, chronic kidney disease; PCI, percutaneous coronary intervention; NYHA, New York Heart Association; LAAC, left atrial appendage closure; OAC, oral anticoagulation; INR, International Normalized Ratio.

### Comparison of MSCT, VR and TEE

**Figure 2:**
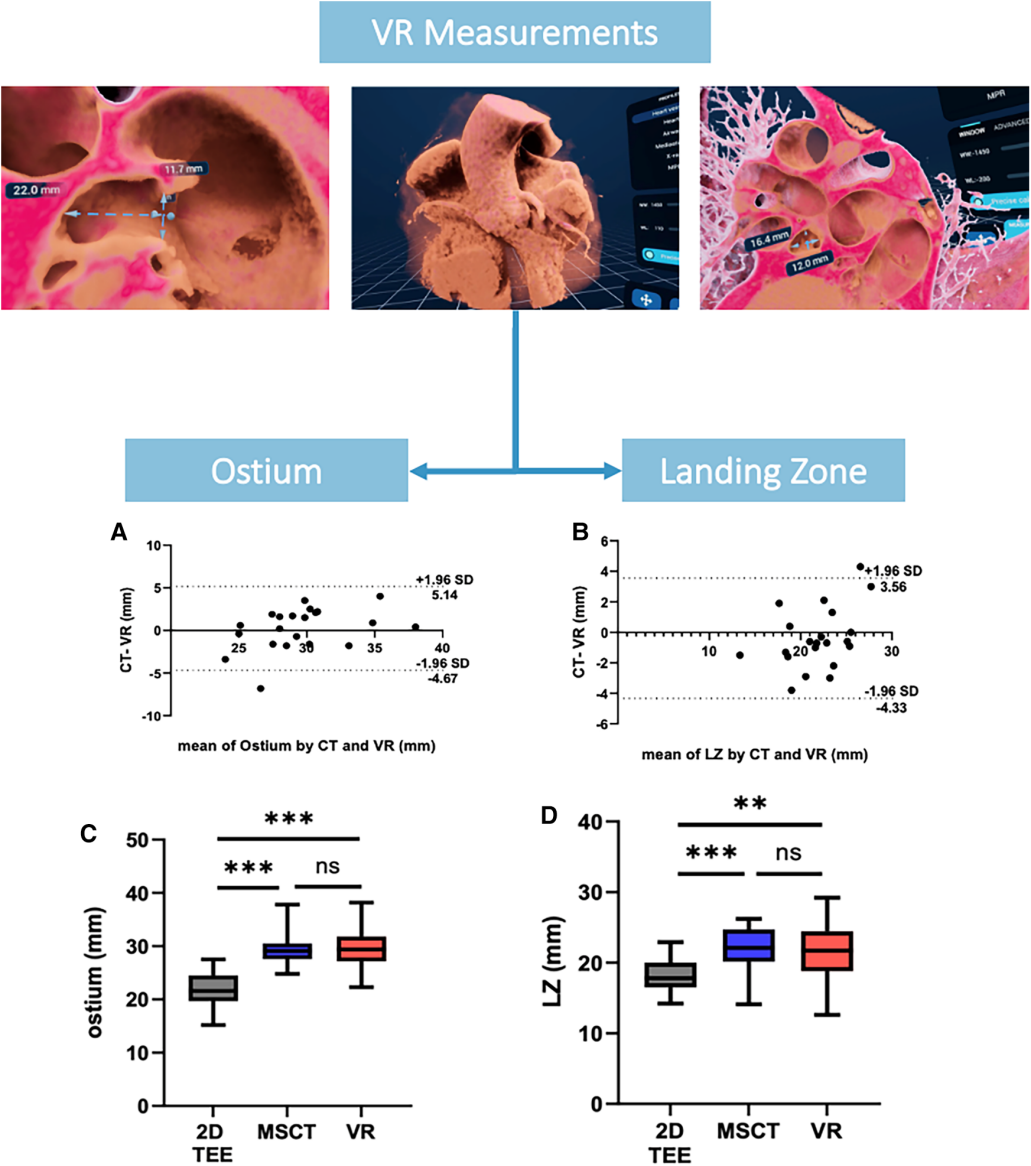
VR measurements of ostium and landing zone. (**A,B**) showing Bland-Altman plots of Ostium and LZ maximum diameters measured by MSCT and VR, (**C,D**) showing an overview of the ostium and LZ diameters derived from 2D TEE, MSCT and VR. VR, virtual reality; MSCT, multi-slice computed tomography; LZ, landing zone; TEE, transesophageal echocardiography.

**Figure 3:**
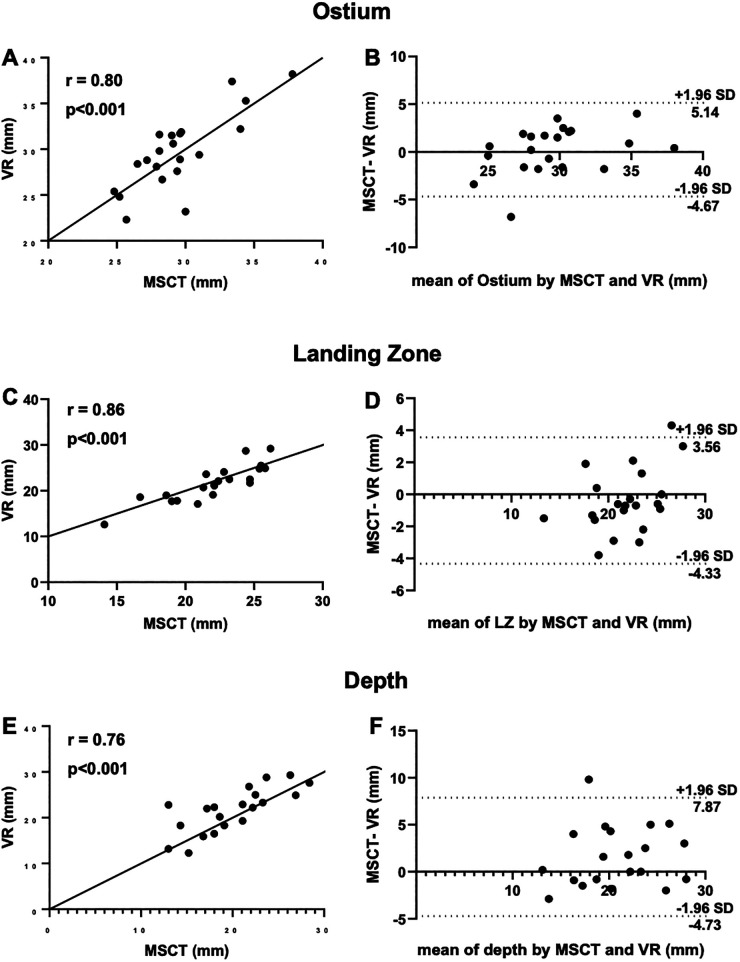
Measurement of ostium, LZ and depth with MSCT and VR. (**A**) Scatterplots showing good correlation of VR measurements of the ostium compared to MSCT; (**B**) Bland-Altman plot of ostium dimensions measured by MSCT and VR; (**C**) good correlation of VR measurements of the landing zone compared to MSCT; (**D**) Bland-Altman plot of ostium dimensions measured by MSCT and VR; (**E**) moderate correlation of VR measurements of depth compared to MSCT; (**F**) Bland-Altman plot of the depth measured by MSCT and VR; CT, computed tomography; VR, virtual reality; LZ, landing zone.

Comparison of mean ostium dimensions between MSCT (29.5 ± 3.2 mm) and VR (29.7 ± 4.1 mm) did not show a significant difference, while measurements in 2D-TEE significantly underestimated ostium diameters (22.2 ± 3.6 mm, *p* = <0.001). Likewise, at the level of the LZ, no significant difference was observed between MSCT (22.0 ± 3.1 mm) and VR measurements (21.7 ± 3.9 mm, *p* = 0.728), whereas 2D-TEE derived dimensions were significantly smaller (18.4 ± 2.65 mm, *p* = <0.001). Lastly, for the LAA depth, no significant difference was observed between MSCT (20.3 ± 4.5 mm), VR (21.6 ± 4.7 mm) and 92D TEE (20.53 ± 4.27 mm, *p* = 0.907; Figures 2C,D). The comparison of MSCT and VR measurements showed good to excellent correlation of Ostium minimum diameter (*r *= 0.93; bias −0.04 ± 1.3 mm), maximum diameter (*r *= 0.8; bias 0.24 ± 2.5 mm) and mean ostium diameter (*r *= 0.88; bias 0.1 ± 1.7 mm; *p* < 0.001). Furthermore, good to excellent correlation was observed at the level of the LZ with the LZ minimum diameter (*r *= 0.84; bias −0.13 ± 1.1 mm; *p* < 0.001), LZ maximum diameter (*r *= 0.86; bias −0.39 ± 2.0 mm; *p* < 0.001) and LZ mean diameter (*r *= 0.9; bias −0.78 ± 1.6 mm; *p* < 0.001). The measurement of the LAA depth showed moderate correlation (*r *= 0.76; bias 1.57 ± 3.2 mm; *p* < 0.001). However, we observed a lower correlation in chicken-wing morphology of the LAA (*r *= 0.63) compared to non-chicken-wing configuration (*r *= 0.87). Figure 3 shows scatterplots and Bland-Altman plots displaying the degree of correlation between MSCT and VR. There was no difference in potential device selection based on VR compared to MSCT in all cases. Table 2 displays detailed information on the comparison of MSCT and VR measurements.

**Table 2:** Comparison between MSCT and VR measurements.

	MSCT	VR	*r*	*p*-value
Ostium min (mm)	21.0 ± 3.4	21.0 ± 3.5	0.93	**<0.001**
Ostium max (mm)	29.5 ± 3.2	29.7 ± 4.1	0.80	**<0.001**
Ostium mean (mm)	25.3 ± 3.1	25.4 ± 3.5	0.87	**<0.001**
LZ min (mm)	18.2 ± 3.1	17.0 ± 3.6	0.94	**<0.001**
LZ max (mm)	22.0 ± 3.1	21.7 ± 3.9	0.86	**<0.001**
LZ mean (mm)	20.1 ± 2.9	19.3 ± 3.6	0.9	**<0.001**
Depth (mm)	20.3 ± 4.5	21.6 ± 4.7	0.76	**<0.001**

Values are presented as mean ± SD. R is the correlation coefficient. *P*-values for the correlation of MSCT and VR measurements are displayed in the table. Significant *P* values (<0.05) are in bold. MSCT, multi slice computed tomography; LZ, landing zone.

### Inter- and intraobserver variability

Measurements of LAA dimensions showed a good to excellent intraobserver agreement for all measurements with ICCs ranging from 0.88 to 0.97. Furthermore, the high reproducibility was demonstrated with a low interobserver variability, with ICCs between 0.90 to 0.98. Table 3 displays ICC values for all LAA dimensions measured.

**Table 3:** Inter- and intraobserver agreement of VR measurements.

	Inter-observer variability (*n* = 21)			Intra-observer variability (*n* = 10)		
	Investigator 1	Investigator 2	ICC (95% CI)	1. Measurement	2. Measurement	ICC (95% CI)
Ostium min (mm)	21.0 ± 3.5	21.3 ± 3.6	0.98 (0.95–0.99)	22.1 ± 3.7	21.2 ± 4.7	0.96 (0.84–0.99)
Ostium max (mm)	29.7 ± 4.1	29.3 ± 3.4	0.93 (0.84–0.97)	31.4 ± 4.5	30 ± 5.9	0.88 (0.59–0.97)
Ostium mean (mm)	25.4 ± 3.5	25.3 ± 3.4	0.96 (0.92–0.99)	26.8 ± 3.7	25.6 ± 5.1	0.92 (0.72–0.98)
LZ min (mm)	17.0 ± 3.6	17.7 ± 3.5	0.95 (0.89–0.98)	18.6 ± 3.9	17.2 ± 4.3	0.92 (0.73–0.98)
LZ max (mm)	21.7 ± 3.9	22.1 ± 3.4	0.95 (0.89–0.98)	23.2 ± 3.7	21.8 ± 3.5	0.88 (0.58–0.97)
LZ mean (mm)	19.3 ± 3.6	19.9 ± 3.4	0.97 (0.93–0.99)	20.9 ± 3.6	19.9 ± 3.8	0.96 (0.83–0.99)
Depth (mm)	21.6 ± 4.7	20.8 ± 4.1	0.9 (0.79–0.96)	23.3 ± 4	20.5 ± 4.4	0.97 (0.87–0.99)

Values are presented as mean ± SD. ICC for measurements for inter- and intraobserver agreement are displayed in the table. ICC, intraclass correlation coefficient; LZ, landing zone.

### Three-dimensional orientation and depth perception

LAA visualization comparing MSCT and VR on a scale 1–5 was judged superior in VR [Median 2 (IQR 1–2)] to MSCT [Median 3 (IQR 2–3), *p* < 0.05].

## Discussion

The main findings of our study are as follows: First, DICOM MSCT imaging data can reliably be visualized in VR. Second, LAA measurements in VR are well correlated to the gold standard MSCT with additional benefit in orientation in the 3D surrounding. Third, measurements in VR showed a high inter- and intraoperator reproducibility.

LAAC is a well-established, safe and effective procedure for stroke prevention in patients with non-valvular AF. Accurate measurements of LAA dimensions are crucial for an optimal device sizing to prevent complications. Numerous comparative studies have shown advantages of MSCT-based LAA sizing and device selection compared to TEE or fluoroscopy. Saw et al. for instance showed a good correlation of ostium and LZ maximal diameter measurements among TEE, MSCT and fluoroscopy, with MSCT providing the largest diameters for ostium and LZ (15). 2D TEE underestimates LZ dimensions up to 20%–40% (16). Another study demonstrated highest accuracy and reproducibility of MSCT measurements compared to other modalities (17). However, in clinical practice volumetric datasets are routinely visualized on two-dimensional screens, which requires cognitive processes and training of the physician for a comprehensive understanding of the three-dimensional anatomy.

Several studies have investigated immersive techniques to overcome this limitation and to fully exploit the capabilities of 3D datasets. Zbronski et al. demonstrated a successful visualization of 3D reconstructed MSCT images in augmented reality before and during LAAC in two patients enhancing the understanding of the patient-specific anatomy (18). Bruckheimer et al*.* demonstrated the applicability of real-time live 3D TEE and 3D rotational angiography during cardiovascular procedures (19). Recently, de Backer et al. have demonstrated that artificial intelligence based MSCT-computational simulation improved device sizing and thereby led to better procedural outcome.

However, VR is a promising innovative, intuitive technology offering the opportunity to fully immerse into a patient-specific 3D virtual environment enabling better spatial perception and orientation for procedural planning. The additive value of visualization in VR was demonstrated in various medical fields. Lu et al. for instance demonstrated a beneficial impact of stereoscopic 3D visualization in patients with congenital heart disease. They were able to show that preoperative 3D visualization of echocardiography, magnetic resonance imaging and MSCT datasets by imaging specialists and surgeons led to the correct diagnosis and supported surgeons in planning the procedure especially by improving the understanding of aortopulmonary collaterals, atrioventricular valve, and pulmonary vein anatomy (20). Likewise, Abjigitova et al. demonstrated an improved understanding of the patient-specific anatomy by the preoperative use of VR in the setting of aortic surgery, leading to a change of surgical strategy in one third of patients (21). A randomized controlled trial even demonstrated a reduction of operative time, blood loss, clamp time and length of hospital stay in the setting of robotic-assisted partial nephrectomy in the VR group compared to conventional surgical planning alone (22). However, little is known about the applicability of VR-based planning for LAAC. Medina et al. (2) evaluated the application of a VR platform for LAAC and revealed a better understanding of the 3D LAA anatomy due to an improved depth perception and free interaction with the virtual models highlighting the potential benefit in training for LAAC. Mill et al*.* (4) analyzed the utility of different computer-based technologies for preprocedural planning of LAAC. The authors underlined different strengths of each modality. While 3D printing was best for understanding the shape of the LAA, a web-based 3D platform was overall best rated offering a detailed visualization of LAA anatomy with the possibility to interact with the 3D models. Here, participants reviewed the images on 2D screens using a standard multiplanar reconstruction. VR, on the other hand, was superior in 3D understanding of the LAA anatomy due to its strength in depth perception. However, the VR application used by the authors enabled interaction with the 3D model and placement of virtual LAA occluder, but did not offer the opportunity to perform measurements in the virtual environment. In a cohort of thoracoscopic LAA closure patients, Van Schaagen et al*.* demonstrated no significant difference between VR and MSCT at the base level of the LAA (23). Tejman-Yarden et al*.* evaluated VR compared to TEE and MSCT in the performance of measurements at the level of the ostium prior to LAAC. They found that maximal ostium diameter measured in VR best predicted inserted device size (24). However, in the case of Amplatzer Amulet™ LAA occluder (Abbott, Illinois, United States), LZ dimensions are decisive for device choice in order to ensure a stable device position. The present work is, to our knowledge, the first to investigate the applicability of VR-based measurements for device sizing at all levels of the LAA. First, the measurements were performed intuitively and precisely in the virtual space and plane. Furthermore, we were able to demonstrate a good to excellent correlation of VR measurements of the ostium with measurements in multiplanar reconstruction in MSCT. Similarly, we could demonstrate a good to excellent agreement of LZ dimension compared to MSCT with good reproducibility. Similarly, depth measurements were performed precisely with a moderate correlation with MSCT with higher correlation in non-chicken wing morphology compared to chicken wing configuration.

Recent studies have shown that MSCT may be the most accurate imaging modality for device sizing for LAAC (25). Considering the improved three-dimensional understanding of the anatomy as well as the good to excellent correlation between MSCT and VR measurements demonstrated in the present study, VR may further add 3D anatomic understanding to measurement accuracy of MSCT datasets. This may potentially influence procedural duration, procedural success and even complications and patient outcomes of LAAC. These questions should be addressed in future studies.

### Study limitations

Our study has some limitations. First, this is a retrospective study with a small number of patients with potential influence of unknown confounders and selection or referral biases. A prospective study is necessary to evaluate potential impact on procedural parameters (i.e., procedural time, contrast and radiation dose) and complication rates. Since no device related complications occurred in the present study, the hypothetical impact of sizing in VR on these complications could not be analyzed. This should be addressed in further studies. Also, only patients with uncomplicated and completed LAAC were included and patients unsuitable for LAAC were not considered. All included patients received an Amulet Amplatzer^™^ device. Other devices such as the Watchman^™^ LAAC device were not included due to the differing sizing algorithm. VR measurements were compared to MSCT and TEE. Other modalities such as 3D printing and 3D TEE were not considered.

## Conclusion

Virtual reality visualization of the left atrium and appendage based on multislice CT data is feasible and allows precise and reproducible measurements in planning of occlusion procedures. Thus, VR offers the benefits of a true 3D visualization with superior depth perception without having to compromise on the precision of the measurements. However, further studies need to explore additional benefits of three-dimensional visualization for operators in preprocedural planning.

## Data Availability

The original contributions presented in the study are included in the article/**Supplementary Material**, further inquiries can be directed to the corresponding author.
