# Supplementary material for: Left atrial appendage sizing for percutaneous closure in virtual reality—a feasibility study
Source: Front Cardiovasc Med. 2023 Sep 1;10:1188571. doi: 10.3389/fcvm.2023.1188571 (PMC10506402; doi:10.3389/fcvm.2023.1188571)
Supplement: Supplementary file 1 [file Table1.pdf]

Table 1: Periprocedural Complications

**Complications (n=21)**	
Pericardial effusion	0 (0%)
Major bleeding	2 (9.5 %)
Minor bleeding	3 (14.3 %)
Major vascular complication	0 (0 %)
Minor vascular complication	0 (0 %)
Stroke (hemorrhagic/ischemic)	0 (0 %)
Device dislocation	0 (0 %)
Arrhythmia	1 (4.8 %)
Thrombus	0 (0 %)
